# Is Your Child’s Face Beautiful?

**Published:** 1909-01-15

**Authors:** Henry C. Ferris

**Affiliations:** Brooklyn, N. Y.


### "IS YOUR CHILD'S FACE BEAUTIFUL.?

BY HENRY C. FERRIS, D.D.S., BROOKLYN, N. Y.

Has your child a beautiful face? No? Why Not? *You* are a good looking man, and your wife is beautiful; why should your offspring be uncomely?

You say that you are not good looking and your wife is not beautiful, but you have in your collection the pictures of your grandparents, who were handsome. They belonged to the strong type, their faces bespeaking character, determination, and intelligence. *Is your type degenerating*, or is your physical condition due to environment and neglect of nature's laws? You may have been better educated than your grandparents, but you have a dejected countenance which belies your intelligence. Is there a commercial value in a face? Ask the man who employs you!—ask the man who is looking for a wife!

To what freak of nature can we attribute these conditions? If we supply an answer, the truth of which can be proved beyond doubt by many experiments, will you listen to our suggestion of a remedy?

Your child breathes through his mouth, and his teeth are not in normal occlusion. You are aware of these conditions and still you allow this perversion of functions to exist until the child grows to be twelve years of age, if he lives long enough, and then you awake to the realization of the fact that something is wrong. Nature has spent more than half of the time allotted to her for developing the osseous structure of the child's face, and then you expect to readjust this organism to render it serviceable with the aid of your mechanical ability!

You regulate the teeth, extract two or three, and though you somewhat reduce the deformity and gruesome expression of the face you are dissatisfied with the result, because nature's architectural plan has been spoiled—the features do not harmonize with the rest of the head, and the balance is imperfect.

If you would but study the growth of the facial bones of your child and understand the forces which produce this development, you could not help appreciating the necessity of assisting nature at an early date by bringing these forces into their proper influence. Remove the obstructions from the nose of your child in his fourth year or earlier; see that the faucial tonsils are not abnormally large, and study the normal occlusion of the inclined planes of the deciduous teeth; watch the locking of the first permanent molars, and if they do not occlude properly, have them placed in their normal position. That a normal occlusion does exist in all types is a fact that has been accepted by the scientists for the last twenty years. If you do not understand this normal occlusion, familiarize yourself with the manner in which the inclined planes of the human teeth occlude. Learn how man masticates his food.

One inclined plane in malposition, one rotated tooth, one abnormal restoration by means of a poor filling, or one extraction, is sufficient to spoil a face which might otherwise show harmonious lines. Do not feed your child with predigested food—with hash and croquettes—after these imperfections have been corrected, but make him chew hard bread-crusts, round steak, etc. In other words, compel the child to exercise the muscles of mastication as you do the muscles of locomotion, thereby improving his digestion and consequent nourishment.

If the muscles are developed, the strain exerted by them upon the supporting osseous parts will necessarily develop structure. If the muscles do not perform their normal functions, however, the osseous structures will undergo abnormal development. If you would have your child grow to be a runner, you would not keep him indoors for a month before a race, and what is true of one part of the system is true of another.

The mother takes her child to a professional man, calling his attention to the child's crooked teeth. The instinct of the parent precedes science. But how many guardians have been put off with the statement that the twelfth year is the proper time for adjusting malformations, and that the nervous strain connected with such correction would be detrimental to the child's health? If an animal cannot masticate its food, will it thrive?

The harmfulness of such advice is elucidated by the fact that by leaving the child's teeth in malocclusion the normal development of the jaw-bone is prevented. Considering, then, that each jaw-bone stands in close relationship to nine bones of the head, it is evident that the whole inclosure of the brain is being retarded in its development. Men need not be scientists to understand what influence this condition will have on a child's nervous system. As soon as this strained condition is relieved by removing the teeth to their normal position, thereby permitting the bones to develop freely, the child grows in weight, as statistics show. Common sense tells us that if a child's arm be put into a splint for six years, atrophy of the osseous and muscular tissues will ensue, and a nervous tension of the whole system be produced. With the removal of this splint the child's nervous system is immediately relieved.

But even then the child's one arm will never attain the proportions of his other, for too large a portion of the period during which the tissues develop has been lost.

This illustration only partly emphasizes the former statement, that the development of the osseous inclosure of the brain which is the citadel of control of the organs, needs the most careful consideration. If you would have your child look beautiful, his brain must not be confined within too small a space, as the face is but the window of intelligence, or the mirror of man's soul. Any imperfection of the system is portrayed in the face. Therefore, the correct functioning of the muscles, having a crushing strength of one hundred pounds or more, in their influence upon man's most powerful organ, the brain, must be carefully observed.

Every part of the dental anatomy, the cusps, the fissures, and the occlusion must be preserved as nature intended them. If one tooth of a set of geared wheels be broken, the engineer would immediately have it replaced to restore the efficiency of his machine. Why should the masticating apparatus be neglected? This principle of preservation does not pertain to orthodontia alone, but to the general practice of dentistry.

Do not extract either the deciduous or the permanent teeth. See that each is in its normal position and that the one hundred and thirty-four inclined planes are performing their natural functions.

Only then a child can develop perfectly and exhibit the highest standard of beauty that is inherent to his type. —Dec. '08. *Cosmos.*

